# How do laypersons interpret the Authentic and Hubristic Pride Scales? A three-study content validity investigation

**DOI:** 10.3389/fpsyg.2026.1699326

**Published:** 2026-02-25

**Authors:** Leah R. Dickens, Brett A. Murphy, Fred Duong

**Affiliations:** 1 Department of Psychology, Kenyon College, Gambier, OH, United States; 2 Department of Health & Behavioral Sciences, Texas A&M University—San Antonio, San Antonio, TX, United States; 3 Independent Researcher, Kalamazoo, MI, United States

**Keywords:** affect, authentic pride, emotion, hubristic pride, measurement, pride, response process validity

## Abstract

The two-facet model of pride posits that the emotion of pride comes in two distinguishable forms: authentic pride and hubristic pride. To date, most empirical research in this vein has relied on the Authentic and Hubristic Pride Scales. Yet, there has been vigorous debate as to whether these scales, particularly the Hubristic Pride scale, are valid as measures of pride. At present, this debate hinges on a question for which there has not yet been empirical evidence sufficient to draw conclusions: do laypersons perceive and interpret these scales as assessing “pride” *as they understand that lay emotion term*? In three studies (total *N* = 509), conducted with native English speakers living in primarily western cultures during summer 2022, we investigate layperson interpretations of these scales across multiple levels of analysis: layperson interpretations of the scales holistically, layperson interpretations of the scale items individually, and layperson free descriptions applying the AP and HP items to their own recalled experiences. Our results indicate that, much as scholars widely disagree as to the meaning of the Hubristic Pride scale, so too do laypersons. These lay interpretations are diffuse, lacking convergence on any single kind of construct. We conclude that layperson interpretations of the Hubristic Pride scale are so extensively varying that its scores probably cannot be used to validly assess pride or any other specific psychological construct.

## Introduction

In recent decades, psychological research on the emotion of pride has been dominated by Tracy and Robins’ (2004, 2007) two-facet model of pride, which posits that the emotion of pride comes in two distinguishable forms: authentic pride and hubristic pride. This model has been extensively empirically investigated using the Authentic Pride (AP) and Hubristic Pride (HP) Scales created by Tracy and Robins (2007; see Table 1 for items). However, as evidenced in a recent exchange in *Emotion* (Dickens and Murphy, 2023; Murphy and Dickens, 2023; Tracy et al., 2023), there is debate as to whether these scales validly measure a two-facet model of pride (also see Kusano and Kemmelmeier, 2022), with Dickens and Murphy arguing that the HP scale’s lack of validity is severe enough that most extant literature on hubristic pride should be rewound. The main research question motivating the current paper is to explore whether laypersons perceive the AP and HP scales as measuring *pride*.

**Table 1:** Authentic pride (AP) and hubristic pride (HP) scale items (Tracy and Robins, 2007).

Indicate the extent to which you feel this way…	
Authentic pride	Hubristic pride
Accomplished	Arrogant
Like I am achieving	Conceited
Confident	Egotistical
Fulfilled	Pompous
Productive	Smug
Like I have self-worth	Snobbish
Successful	Stuck-Up

Prior critiques (Dickens and Murphy, 2023; Holbrook et al., 2014a, 2014b) have disputed Tracy and Robins’ (2007) claim that the AP scale captures pride *when one attributes success to one’s hard work* and the HP scale captures pride *when one attributes success to one’s stable characteristics* (e.g., being intellectually brilliant, naturally athletically gifted, etc.). Although these two attributional pathways were central to Tracy’s and Robins’ original model of authentic and hubristic pride (Tracy and Robins’, 2004, 2007), and based at least in part on parallels with guilt and shame, Tracy et al. (2023, see ps. 894–895)^1^ seemed to have removed this attributional perspective from their model of pride or have at least reduced its centrality (e.g., Tracy et al., n.d.). Nonetheless, they still maintain that both scales validly measure pride; as they see it, any limitations may be in their original theoretical model rather than in the scales themselves (Tracy et al., 2023). Moreover, whereas some have argued that the HP scale is not a measure of pride at all, because it facially and empirically does not align with scholarly *scientific* conceptions of pride as an emotion (Dickens and Murphy, 2023; Holbrook et al., 2014a, 2014b), Tracy and Robins (2014) and Tracy et al. (2023) continue to defend it because they claim it aligns with how *laypersons* themselves define and describe “pride.”^2^

To emphasize: Tracy and colleagues do not place importance on whether the AP/HP scales reflect any technical scholarly scientific definition of pride. Instead, they now view the validity of the scales as founded on whether or not they capture what the vernacular word “pride” means to laypersons, as they experience it as a first-person emotion in moments in everyday life.

At present, the scientific value of the HP scale (and the extensive data collected using it) hinges on the question: do laypersons understand the HP scale as measuring pride? Although a great deal of empirical evidence has been presented in debates about other aspects of the scales’ validities, little or no empirical data has thus far been available to help adjudicate whether laypersons do indeed interpret the HP (and AP) scales as capturing “pride” as laypersons understand that term. This article aims to provide such data.

### Why do Tracy and Robins believe their scales capture layperson understandings of pride?

In constructing the AP and HP scales, Tracy and Robins took a “bottom-up” approach in which they sought to let the items for the scales emerge from layperson descriptions and ratings of (a) the nonverbal pride expressions of others and (b) their own recalled pride experiences, as well as via other methods, such as having laypersons provide prototypicality ratings for different pride-related words. As a result, they have argued, “Clearly, lay people view the scale items as representative of pride, given that they provided the list of words related to pride that now constitute the scale items” (Tracy et al., 2023, p. 896).

Dickens and Murphy (2023), however, speculated that the various methods Tracy and Robins took in creating the scales inadvertently created an HP scale which, rather than capturing pride, instead captures a mystery stew of different language meanings and uses, some far removed from the emotional experience of pride. For example, Dickens and Murphy theorized that many heightened scores on the HP scale might reflect, not a first-person experience of the emotion of pride, but, rather, an awareness that *others* have judged oneself to be pretentious, arrogant, etc. (for similar concerns, see Holbrook et al., 2014a, 2014b). Tracy et al. (2023, p. 896) acknowledged that such a concern “may very well be true, but no [empirical] evidence exists to support it.” More broadly, “without new studies probing into participants’ cognitive processes when they complete the scale (a research direction Dickens and Murphy suggested), all we can know is that the HP scale items came from participants’ reports of actual pride experiences…” (Tracy et al., 2023, p. 897).

Reviewing the methods in Tracy and Robins (2007), Dickens and Murphy disputed that the HP scale items corresponded to participants’ first-person experiences of their own emotions of pride. For example, it is unclear whether these items reflect a first-person (“I feel snobbish”) rather than a third-person (“That person seems snobbish”) experience of pride or a cognitive evaluation that others might be perceiving one in a negative light (“They probably think I am snobbish”) (for extensive discussion, see Dickens and Murphy, 2023). Even if all of these scale items do emerge from first-person experiences of pride, though, that would not guarantee that the final scales would be recognized by laypersons as capturing “pride.”^3^ In sum, the question is whether laypersons, when presented with these scales (particularly the HP scale), will understand them as reflecting first-person emotional experiences of pride. If they do not, then the final defense of the HP scale’s validity will no longer hold.

## Overview of the current studies

Do laypersons understand the AP and HP scales as both capturing “pride,” at least as those laypersons understand that concept term? Though this kind of question is quite unusual in the psychological assessment literature (because it is rare for researchers to couch validity in terms of fidelity to lay meanings of concept terms), it can be understood as pertaining to both *content validity* and *response process validity* (response process validity can be understood as an aspect of construct validity, Bandalos, 2018).

An assessment’s scores have content validity if the test items validly represent the intended content domain. A common method for achieving content validity is to recruit a panel of scholars to evaluate and rate the validity of the items (e.g., as in Zielinski and Veilleux, 2018). Given that Tracy and colleagues view laypersons as the proper experts in this case, laypersons can serve as the content validity panel.

An assessment’s scores have response process validity if the cognitive processes occurring in the test-taker while completing the assessment align with what the test developer intends (e.g., are test-takers interpreting the items the way the test developer intends?).^4^ Although investigations of response process validity have thus far been rare in the psychological assessment literature, especially past the earliest stages of measure development, researchers have emphasized the need for greater efforts in this area (e.g., Boness and Sher, 2020), such as “comparing how non-expert and expert (i.e., researcher) participants interpret items on popular psychology measures” (Mason et al., 2023, p. 23). The most common method for achieving response process validity is to conduct interviews with a small number of participants (for discussion of rationale and different interview methods, see Peterson et al., 2017), but response process issues have also been investigated via survey methods (e.g., Turner, 2014), eye tracking (e.g., Yaneva et al., 2022), keystroke logs (Davidson, 2022), and other methods.

In the case of the AP and HP scales, Tracy et al.’s defense of them as capturing layperson perceptions of pride prompts us to ask a broad question: do test-takers understand and respond to the scales and their items as reflecting “pride,” as Tracy et al. presume and intend? This broad question has both content validity and response process validity elements, but they are intertwined and we do not attempt to strongly separate them in this article.

To investigate this question, we conducted three studies. In the first study, we presented layperson participants with the full AP and HP scales and queried them about their perceptions of each of the scales holistically. In the second study, we presented layperson participants with the 14 individual items from the scales and queried them about their perceptions of each item. In the third study, we prompted participants with terms from the AP and HP scales and asked them to recall and describe a time when they felt that way; this data allowed us to better understand what participants are doing when they try to apply such items to their own experiences.

As is typical of content validity and response process validity investigations (as well as of investigations of lay prototypes of emotions, e.g., Fehr and Russell, 1991), the primary data of interest collected in our studies is descriptive and requires interpretive judgment. For example, what percentage of participants must rate the HP scale as highly reflective of “pride” for us to consider it as validly capturing a layperson understanding of pride? Much as with other evaluations of content and construct validity (e.g., How strongly should the panel of scholarly raters agree about an item’s validity to retain it?), there are no universal thresholds. Rather than imposing arbitrary cutoffs by which we would mechanically make judgments, we draw conclusions based on the totality of patterns that converged across the three studies. We encourage readers to examine the variable distributions we provide and independently evaluate our conclusions.^5^

### Transparency and openness

All study demographics are provided in Table 2. All studies were approved by the institutional review board at Kenyon College and then were conducted on Prolific, with native English speakers, during summer 2022. We aimed to recruit a sufficient number of participants to allow us to make reasonable descriptive estimates. Only participants who signed the electronic informed consent—presented on the first page of the online survey—were allowed to move forward with the study. Only native English speakers were included in analyses; despite limiting Prolific parameters to native speakers, we removed seven participants from Study 3 who self-reported as non-native English speakers.

**Table 2:** Demographic information for the study samples.

Characteristic	Study 1: full scales (*N* = 147)	Study 2: single items (*N* = 154)	Study 3: writing prompts (*N* = 208)
Gender			
Men	56	60	82
Women	88	89	120
Other/No response	3	5	6
Age M (SD) in years	35.3 (13.6)	35.0 (13.1)	33.1 (12.6)
Race/ethnicity			
White/Caucasian	122	115	144
Black/African descent	10	20	28
Asian/Asian descent	6	9	11
Multiracial	1	3	17
Other/No response	8	7	8
Location			
US and Canada	58	68	137
UK and Ireland	70	65	57
South Africa	8	13	14
Other	6	5	0
No response	5	3	0

*N* represents the full analyzed sample size, after excluding participants for low data quality. For Study 3, these numbers reflect unique participants who answered prompts only regarding their own personal experiences; some of them provided data for both AP and HP experiences; we excluded participants who wrote only third-party accounts of AP/HP (resulting in the removal of 62 participants who supplied no first-person data).

Data quality screening was conducted in four ways (for exemplar of our approach, see Douglas et al., 2023). First, we checked for any repeated IP addresses or Prolific identification numbers; we found none. Second, we excluded participants who failed one or more attention checks in the survey; this resulted in the removal of one participant from Study 1, five from Study 2, and five from Study 3. Third, anyone who provided nonsensical or missing data to free-response questions was removed from the analyses of free-response data. For instance, people who simply typed a numerical digit, rather than a text answer, were excluded; this excluded three participants from Study 1 and one from Study 2. Finally, we determined the minimum amount of time required to complete the full survey by timing four research assistants, who were instructed to take the survey and finish as quickly as possible while still reading and answering all questions honestly. We calculated their average time and standard deviation, and removed any participants who fell below one SD of the mean. This resulted in the exclusion of two participants in Study 1 (completing in under 5 min) and one in Study 2 (completing in under 4 min).

The full datasets for all three studies, including both de-identified participant data and the research assistant codes of the qualitative participant responses, are available at: https://osf.io/7sf6x/?view_only=fa645c24b28441c6bc10bff5d92f247c. Data were primarily analyzed using SPSS version 28.01.1.1 and R version 4.3.0. These studies were not preregistered; our analyses are exploratory. Full Qualtrics surveys are available at the above link; many variables in these surveys were not directly relevant to our main response process validity questions, and we do not discuss them here.^6^

### Study 1 (full scales)

Study 1 investigated the full AP scale *holistically* and the full HP scale *holistically*. Participants were presented with one full scale and then the other, in random order, and asked to provide an open-ended description as to what they thought the scale was measuring (see Table 3 for exact wording of open-ended questions across studies). In addition to this free response data, we then also asked participants to rate the scales in relation to specific components of the consensus scholarly definition of pride construct (e.g., “Do you think this scale measures something related to success?”; see Table 4 for exact wording of the rating questions). Finally, at the end, we presented them with the consensus scholarly definition of pride (see Table 4) and asked them whether they believed the scale captured pride. This final step does not mean that we insist that layperson understandings of the AP/HP scales must align with scholarly definition of the term; instead, we use the scholarly definition components merely as a vehicle to explore layperson understandings. In sum, this study illuminated how laypersons interpret the meaning of each scale *as a whole*.

**Table 3:** Free response question wordings across studies.

Study	Question title	Instructions
Study 1 (full scales)	Scale description	“From just this information alone, what do you think this scale is measuring? You are welcome to use one word or many to explain your thoughts.”
Study 2 (single items)	Item description	“For each item, please imagine you strongly feel this way right now. Without using the given word, describe the emotion you would likely be feeling in this moment. You may choose different words or the same word for each, as you think appropriate. What emotion would you likely be feeling for each item?”
Study 3 (writing prompt)	AP experience	“‘Right now, in this current moment, I am feeling successful, accomplished, and confident.’ Can you remember a moment when you felt this way? Please tell us a little bit about that moment when you felt this way. What happened? Try to describe the experience in a few sentences.”
	HP experience	“‘Right now, in this current moment, I am feeling pompous, conceited, and arrogant.’ Can you remember a moment when you felt this way? Please tell us a little bit about that moment when you felt this way. What happened? Try to describe the experience in a few sentences.”
	AP cause	“Why did you feel successful, accomplished, and confident?”
	HP cause	“Why did you feel pompous, conceited, and arrogant?”
	AP/HP other emotions	“What other emotions were you feeling in that moment? Please try to tell us as much about your emotions in that moment as you can remember.”

**Table 4:** Rating Question Wordings aand Response Options

**Study**	**Question Title**	**Instructions**		**Scale**
Study 1 (Full Scales)	*Emotion State?*	“Do you think this scale is measuring an emotional state?”		1 = *Definitely Not* to 5 = *Definitely Yes*
	*Emotion Valence?*	“If someone endorsed these items (scoring high on this scale; "Extremely"), do you think they would feel good or bad about feeling that way?”		1 = *Extremely Bad* to 7 = *Extremely Good*
	*Success-Related?*	“Do you think this scale measures something related to success?”		1 = *Definitely Not* to 5 = *Definitely Yes*
	*Align with Pride Definition?*	“Pride, as an emotion, can be defined as, ‘a positively valenced emotion that occurs in response to success.’ Positively valenced means it is a pleasurable emotion to feel. With this definition in mind, how likely is it that the scale is measuring pride?”		1 = *Not at all Likely* to 5 = *Extremely Likely*
Study 2 (Single Items)	*Emotion State?*	“Do you think each item is related to an emotional state?”		1 = *Definitely Not* to 5 = *Definitely Yes*
	*Emotion Valence?*	“Imagine you strongly feel each of these items right now. For each item, please consider whether you would feel good or bad about feeling that way.”		1 = *Extremely Bad* to 7 = *Extremely Good*
	*Success-Related?*	“To what degree would you say each item sounds related to success?”		1 *= Not at all Related* to 5 *= Extremely Related*
	*Align with Pride Definition?*	“For each item, please consider whether you think that this item might be relevant to the emotional experience of pride, which can be defined as ‘a positively valenced emotion that occurs in response to success.’ Positively valenced means it is a pleasurable emotion to feel. How relevant is each item to this definition of pride?”		1= *Not at all Relevant* to 5= *Extremely Relevant*
Study 3 (Writing Prompt)	*Emotion Valence?*	“When you felt that way, how were you feeling in general?” (Self-Assessment Manikin)		1 = *Very Unpleasant* to 9 = *Very Pleasant*
	*Emotion Items?*	“Were you feeling any of these other emotions during that moment? (happy, content, good, bad, sad, upset, embarrassed, ashamed, guilty, proud of my hard work, proud of my talents, respected by others, disliked by others, self-conscious, angry, irritated)”		*Yes; No; Maybe*
		**General Positive Valence**	**General Negative Valence**	**Negative Self-Conscious Emotions**
		Happy	Bad	Embarrassed
		Content	Sad	Ashamed
		Good	Upset	Guilty
		**Pride**	**Social Evaluation**	**Others**
		Proud of my hard work	Respected by Others	Angry
		Proud of my talents	Disliked by Others Self-Conscious	Irritated

### Study 2 (individual scale items)

Study 2 investigated the full AP and HP scales *on an item-by-item basis*. For each scale item (randomized together), we asked participants to give a free response description of the emotions they would likely be feeling if they endorsed that item. We then asked them to rate how relevant each item related to various components of the scholarly definition of pride, such as whether the item was positively valenced. Finally, we asked them how well each item aligned with the consensus scholarly definition of pride. This study illuminated how laypersons interpret the meaning of *each of the scale items individually*.

### Study 3 (writing prompts)

Study 3 investigated how participants respond to prompts asking them to relive experiences based on a representative subset of the AP scale items and HP scale items. Participants first saw one of two prompts (in random order): (1) “Think about this statement: ‘Right now, in this current moment, I am feeling successful, accomplished, and confident.’ Can you remember a moment when you felt this way?” or (2) “Think about this statement: ‘Right now, in this current moment, I am feeling pompous, conceited and arrogant.’ Can you remember a moment when you felt this way?” For the current work, we only used data from individuals who responded “yes” to the prompt, resulting in 177 AP experiences and 95 HP. For these participants, we asked them to tell us about the moment when they felt this way and to describe the experience. We subsequently asked them (1) what other emotions they were feeling at that moment (both via free response and by rating a list of potential emotions) and (2) why they felt the way they did (free response). This study provided us with data about how laypersons understand these types of scale items when trying to apply them to their own experiences.

## Primary question: Do laypeople interpret both the AP and HP scales as reflecting “pride” *as they understand it*?

The most important question is whether laypersons spontaneously interpret these scales (and individual items) as measuring “*pride.”* We predicted laypeople would view the AP scale – and its individual items – as measuring pride, but not the HP scale and its items. If laypersons do consensually agree that the HP scale reflects a first-person experience of pride, this would alleviate scholarly concerns about the scale’s response process validity. If not, then the final defense of the HP scale will no longer hold. We addressed this question across all three studies.

### Study 1 free response data (scales assessed holistically)

**Figure 1:**
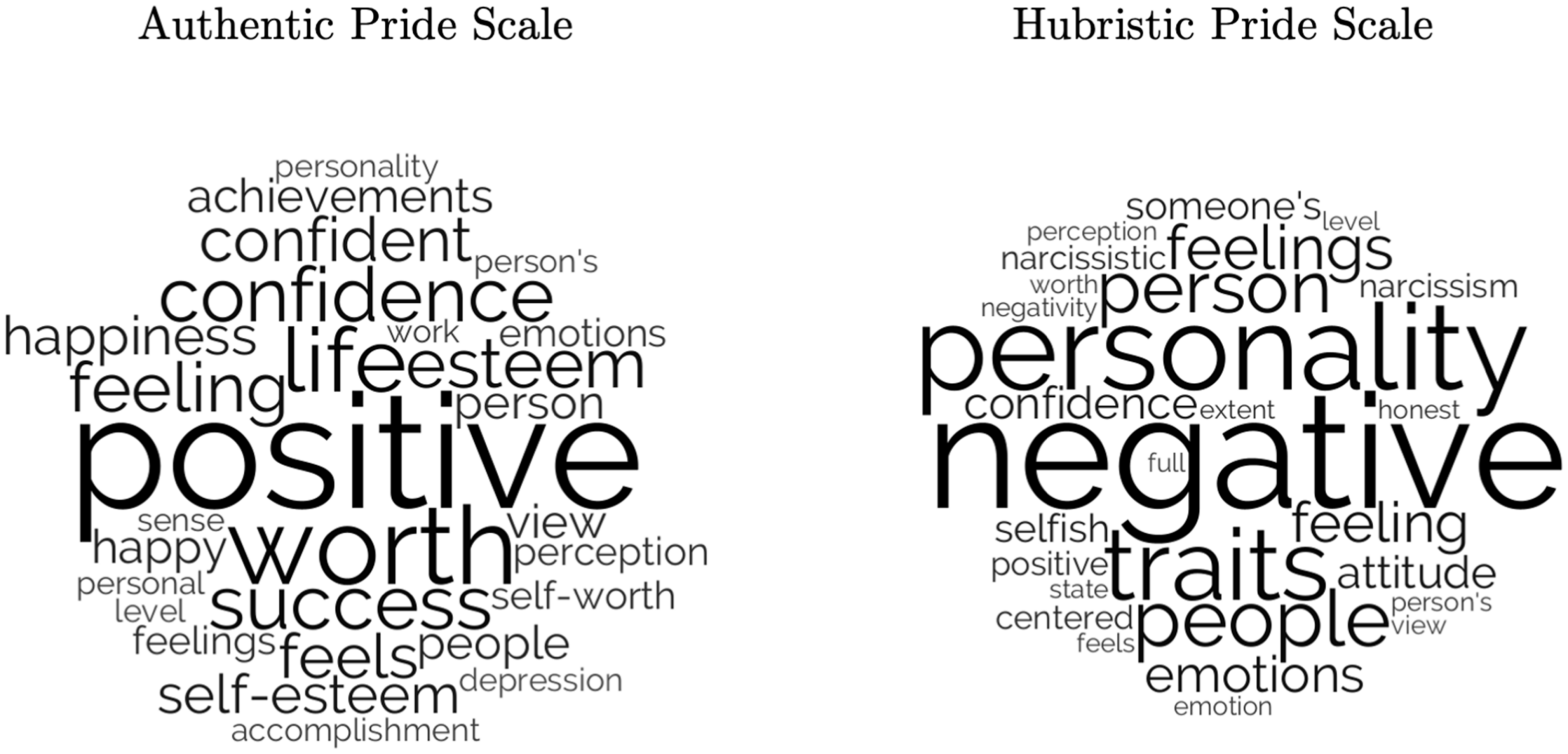
Word clouds of free responses to “What Do You Think This Scale is Measuring?” (study 1). Within each cloud, larger words indicate higher frequency. Word size comparisons across clouds are not indicative of equal frequencies. Word clouds generated with LIWC-22 using default word cloud settings (Boyd et al., 2022).

We showed participants the entire AP scale and the entire HP scale separately (in random order), and directly asked them, “What do you think this scale is measuring?” We noted whether participants used the words “*pride”* or “*proud”*^7^ in their answers. Participants rarely said *pride* or *proud* for either the AP or HP scale. Only three participants (2.1%; 95% CI [0, 4.4%]) used either of these terms to describe the AP scale; two (1.4%; 95% CI [0, 3.0%]) used either of the terms to describe the HP scale. See Figure 1 for a graphical representation of word frequency.

Following the basic process of thematic analysis outlined by Braun and Clarke (2006), we had six undergraduate research assistants, who were blind to the prompting questions, independently code these open responses and categorize them by theme. Then, the six coders, with the supervision of Dickens, reached consensus on relevant themes, resulting in a total of 21 potentially unique themes. Using these new thematic labels, the six coders again worked independently to categorize responses, before at least two coders worked together to agree on thematic categorizations for each response (the number of themes reported per response ranged from one to four)^8^. The complete qualitative coding of the dataset is presented in Table 5.

**Table 5:** Themes of open responses to the question: “What do you think this scale is measuring?” (Study 1).

	AP		HP	
Themes	Frequency	Percentage	Frequency	Percentage
Positive valence themes				
Self-esteem/self-worth	62	44.0%	16	11.3%
Accomplishment/success	33	23.4%	0	
Positive emotions	16	11.4%	0	
Positive attitudes	13	9.2%	0	
Positive traits	10	7.1%	5	3.5%
Satisfaction	8	5.7%	0	
Pride	3	2.1%	2	1.4%
At least one of the above positive themes		*84.3%*		*16.3%*
Neutral themes				
General self-perception/self-awareness	13	9.2%	11	7.8%
General emotions	18	12.8%	17	12.1%
General attitudes	2	1.4%	6	4.3%
General traits	3	2.1%	17	12.1%
Social comparisons	0		10	7.1%
Others’ social judgments	0		6	4.3%
Privilege	0		2	1.4%
At least one of the above neutral themes		*22.9%*		*42.6%*
Negative valence themes				
Negative self-perception	0		14	9.9%
Negative emotions	2	1.4%	12	8.5%
Negative attitudes	0		12	8.5%
Negative traits	0		23	16.3%
Arrogance	0		16	11.3%
Selfishness/self-centeredness	0		17	12.1%
Narcissism	0		10	7.1%
At least one of the above negative themes		*1.4%*		*56.0%*

For AP, the total number of analyzable responses was 140; for HP, 141. Some longer responses fell into more than one theme. If a response specified valence, it was categorized only into positive/negative themes.

Overall, the AP scale was typically characterized by positively valenced themes, with some neutral themes and only two responses with a negatively valenced theme. On the other hand, the responses to the HP scale were mostly negatively valenced themes, along with a significant number of neutral themes; positively valenced themes were much less common. For AP scale descriptions, the most frequently occurring themes were: (1) self-esteem or self-worth (44.0% of responses), (2) success or accomplishment (23.4%), and (3) general emotions (12.8%). For HP scale descriptions, the most frequently occurring themes were: (1) negative traits (16.3%), (2) selfishness or self-centeredness (12.1%), (3) general traits (12.1%), and (4) general emotions (12.1%).^9^

### Study 2 free response data (scales assessed item-by-item)

In Study 2, we showed participants the individual items from the AP and HP scales (in random order), and instructed, “Please imagine you strongly feel this way right now. Without using the given word, describe the emotion you would likely be feeling in this moment…What emotion would you likely be feeling?” Again, we coded whether participant responses contained the words *pride* or *proud*. Participants gave a response for each of the 14 items. 39.3% of participants (95% CI [31.5, 47.0%]) used pride or proud when describing their emotions for at least one of the AP items; 20.3% (95% CI [13.9, 26.6%]) used pride or proud in reference to at least one of the HP items. These percentages seem rather low, but people do think of AP terms as pride-related roughly twice as much as HP terms.

**Figure 2:**
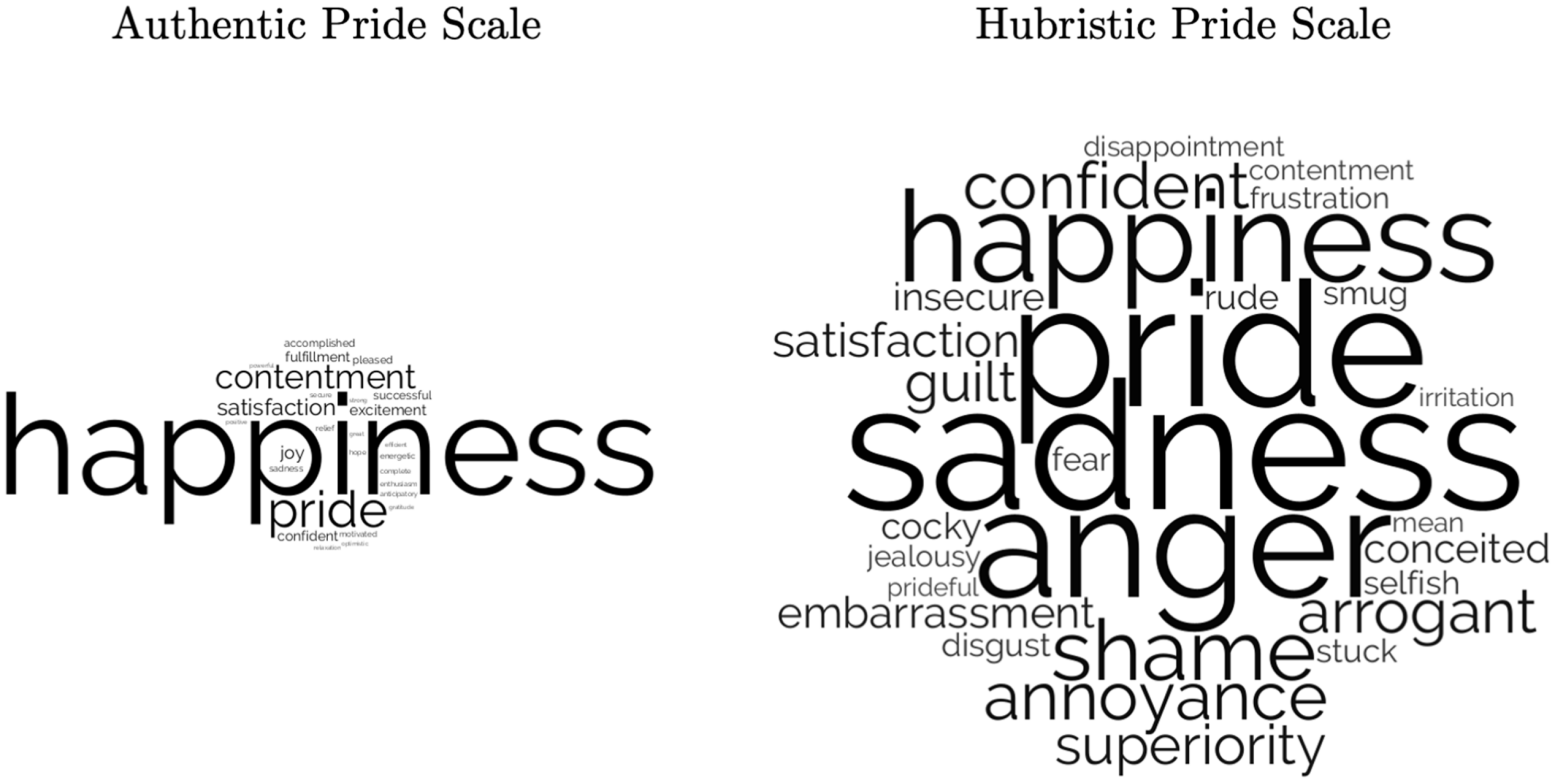
Word clouds of free responses to “What Emotion Would You Likely be Feeling for Each Item?” (Study 2). Within each cloud, larger words indicate higher frequency. Word size comparisons across clouds are not indicative of equal frequencies. Word clouds generated with LIWC-22 using default word cloud settings (Boyd et al., 2022).

We investigated the frequency with which different emotion terms were referenced in these free responses. For our list of emotion terms, we modified (e.g., used all noun/adjective/adverb variations of each term) the list of discrete emotion terms in Table 1 of Weidman et al. (2017; see Supplementary materials for all terms). For the AP items, the five most common emotion terms were: happiness (450), pride (130)^10^, contentment (74), joy (32)^11^, and excitement (26).^12^ For the HP items, the five most common emotion terms were: sadness (90), pride (87)^13^, anger (76), happiness (40), and shame (37).^14^ Responses are depicted in word cloud form in Figure 2, and in table form in Supplementary Table S8.

### Study 3 data (recalling AP/HP experiences)

For Study 3, we asked participants to consider whether they had ever experienced a time when they felt “*successful, accomplished*, and *confident*” (subset of AP items) or a time when they felt “*pompous, conceited,* and *arrogant*” (subset of HP items). Of our full sample of 270 (before removing those who did not write first-person accounts of either AP nor HP), 93.3% of participants could remember a time they experienced AP, but only 35.2% of participants could recall a time they experienced HP.^15^ Participants who did indicate that they could remember such a time were instructed to write about the experience in detail, including to tell us why they felt that way. We then asked, “What other emotions were you feeling in that moment?”.

#### Free response data

We coded the free responses for each participant (including the initial description, the “cause” question, and the emotion free response) and noted whether they spontaneously used the words *pride* or *proud* in at least one of their free responses. For AP responses, 44.7% of participants (95% CI [37.4, 52.0%]) referred to pride; for HP, it was 23.3% of participants (95% CI [14.5, 32.1%]).

#### Rating data

**Figure 3:**
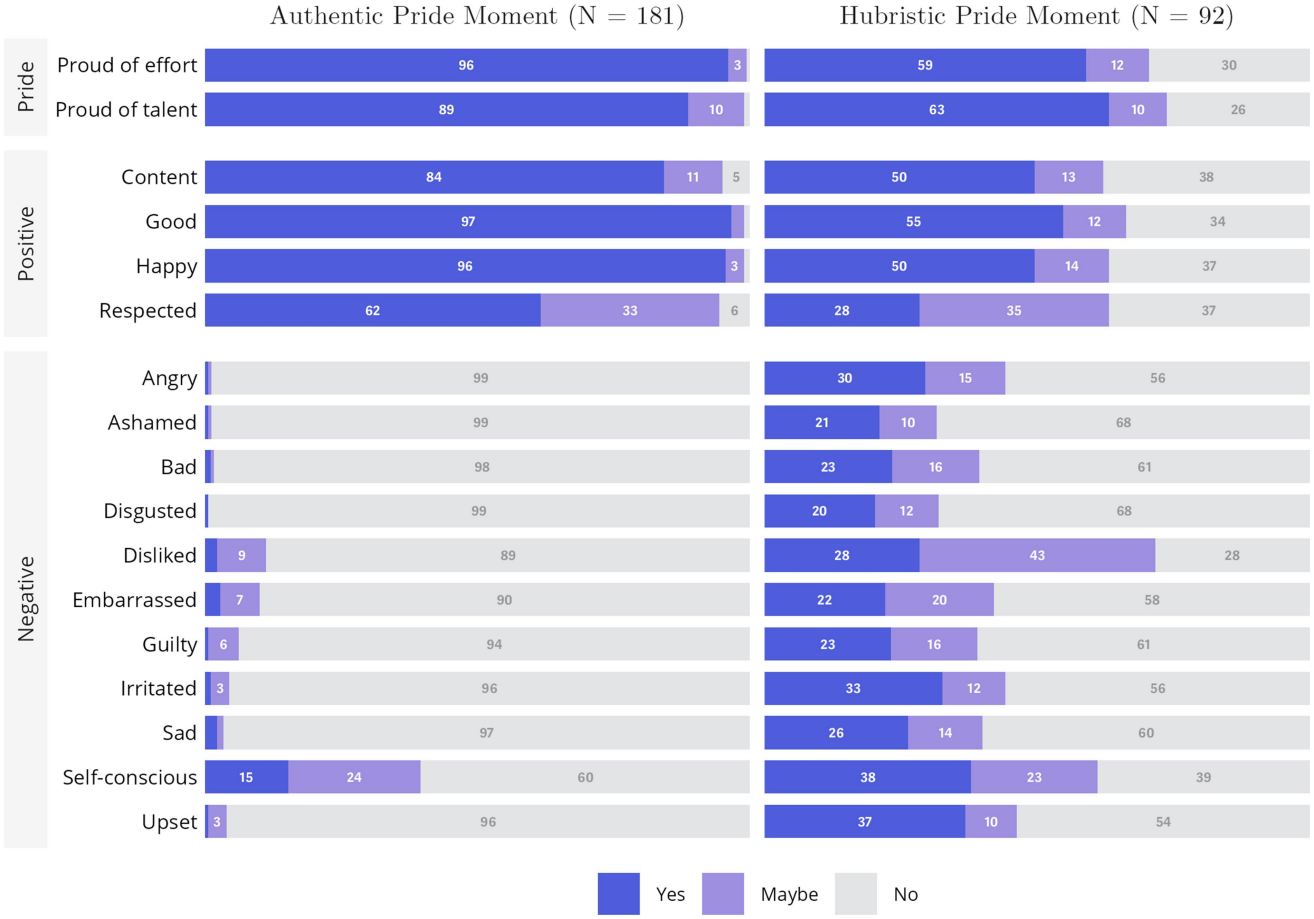
Study 3 ratings of emotions in recalled AP or HP moments. The data presented is in percentages of all responses, not total counts.

We also gave participants a list of emotional responses and asked them to indicate whether they felt any of the listed emotions during their personal experiences of AP and HP (see Table 4 for full list and Figure 3 for a visual representation). Two of the items were “proud of my hard work*”* and “proud of my talents.” For experiences of AP, 87.6% of participants reported “yes” to feeling both in the moment they described to us, whereas only 2.8% of participants did not report (“yes”) feeling either of them. For HP, 51.6% indicated both, whereas 29.5% of participants did not report (“yes”) feeling either of them. A substantial number of HP responses indicated feeling negative self-conscious emotions, such as shame, embarrassment, and guilt; these kinds of emotions are conceptually opposite of pride in most theoretical models (e.g., Tracy and Robins, 2007).

### Primary question synopsis

Taken together, the free response data from Studies 1 and 2 indicate that most laypeople do not *spontaneously* interpret either the AP or HP scales or their items as capturing “pride.” Instead, they appear to typically interpret the AP scale content as capturing a broader conceptual domain of self-esteem, success, and general positive affect. In contrast, layperson perceptions of the HP scale appear much more varied, with some perceiving it as capturing negative character aspects such as narcissism and selfishness, some perceiving it as related to negative emotions like sadness, anger, and shame, and some perceiving it as related to pride.

The story is somewhat different when looking at the rating data from Study 3, where we presented participants with items *explicitly* referencing being “proud.”^16^ For AP experiences, the rating data showed that the overwhelming majority of participants reported feeling “happy,” “proud of their talents,” and “proud of their efforts.” In conjunction with the free response data across the three studies, we conclude the AP content is strongly related to “pride” as laypersons understand that term, but may encompass broader content than just the lay concept of pride.

For HP, the rating data (and the free response data) from Study 3, though, is difficult to interpret because only a minority of participants could recall (or admitted being able to recall) a time when they had HP experiences. Thus, we cannot know whether the data provided for HP experiences are fully generalizable across people or, instead, are particular to the minority of participants who provided them to us.

Of the minority of participants who were able to recall a time when they experienced HP, the rating data indicates that most did feel “proud,” at least when that term was explicitly given as an option, but almost a third of them did not. As will be further seen in this article, we conclude that laypersons have widely varying understandings of the meaning of the HP scale and its items. As a result, and as argued by Dickens and Murphy (2023), scores on the HP scale might reflect so many different response processes that it is difficult to see how the scale could be confidently used to measure any one particular construct, whether “pride” or something else.

## Secondary question: Do laypeople interpret both the AP and HP scales in line with the *consensus scholarly definition of pride*?

In their critique, Dickens and Murphy (2023) systematically argued that the HP scale does not align with the consensus scholarly definition of pride: “Pride is a positively valenced emotion that occurs in response to success” (Mercadante et al., 2021; p. 30, coauthored by Tracy).^17^ Dickens and Murphy tackled each aspect of this definitional scheme in order, arguing that the HP scale did not meet any of the definitional components. Whereas they heavily relied on meta-analytic empirical evidence and conceptual arguments regarding face validity to support their claims, here we ask a simpler series of questions: do layperson understandings of these scales line up with the components of the scholarly scientific definition of pride?

Again, Tracy et al. (2023) do not look to this kind of scientific definition as the basis for the HP scale’s validity; they look to layperson understandings of the word “pride.” Thus, the analyses here are secondary but complementary to those discussed for our primary question: they help illuminate what laypersons do and do not think these scales reflect.

### Aligns with scholarly definition of pride?

The first step in investigating this secondary question domain is to simply present laypersons with the scientific definition of pride and ask them whether they think the AP/HP scales – or their items – align with it.

#### Studies 1 and 2 rating data

**Figure 4:**
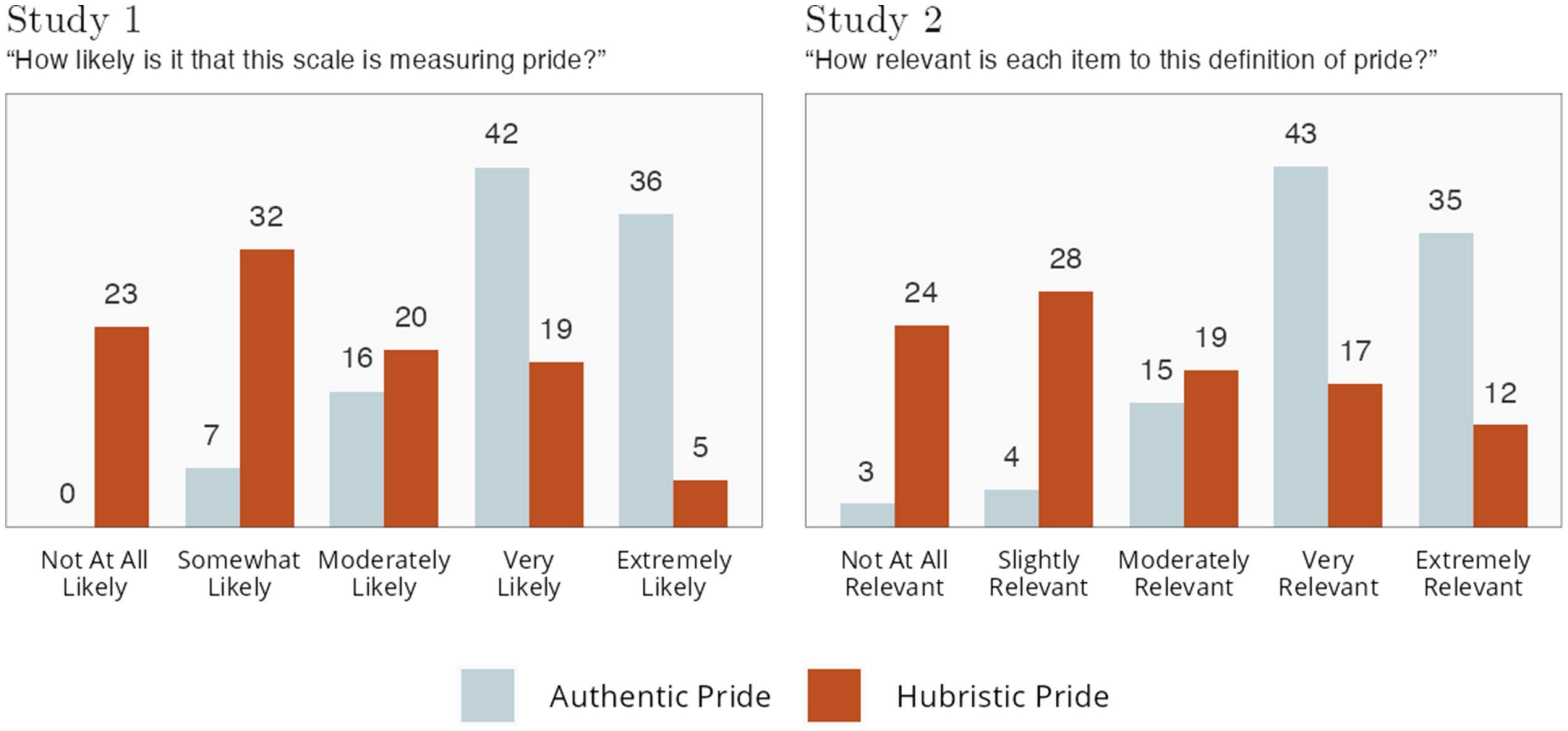
Ratings of alignment with scholarly definition of pride. Numbers indicate percentages.

In Studies 1 and 2, *at the end of the surveys* (so as not to bias their other responses), we showed participants the consensus scholarly definition of pride. Then, we asked how likely it was that each of the two scales (or each of the 14 items) measured pride. Results across both studies can be seen in Figure 4.

#### Conclusion

Our layperson participants very strongly endorsed the AP scale and its items as measuring the scholarly definition of pride; it was very rare for participants to say that the scale or items were “not at all likely” or “not at all relevant” to measure this definition of pride. There was far less layperson consensus regarding the HP scale. Although some participants indicated the HP scale/items were “very” or “extremely” likely/relevant to this definition of pride, roughly as many indicated they were “not at all” likely/relevant.

### Emotion-related?

To further explore our secondary question, *before* directly presenting participants with the full scholarly definition of pride, we queried them regarding each of the conceptual components of that definition of pride: (a) an emotional state; (b) positively emotionally valenced; and (c) related to success. This allowed us to more deeply investigate whether there is a mismatch between layperson interpretations of the scales and the scholarly scientific definition of pride.

We start with whether laypersons perceive the AP and HP scales as measuring an emotional state. Although Dickens and Murphy (2023) argued that the HP scale did not capture an “emotion,” but rather a “cognitive evaluation,” this was one of the weakest parts of that systematic critique, as there was no empirical evidence to support this argument. Here we investigate whether laypersons perceive these scales as capturing an emotional state.

#### Studies 1 and 2 rating data

**Figure 5:**
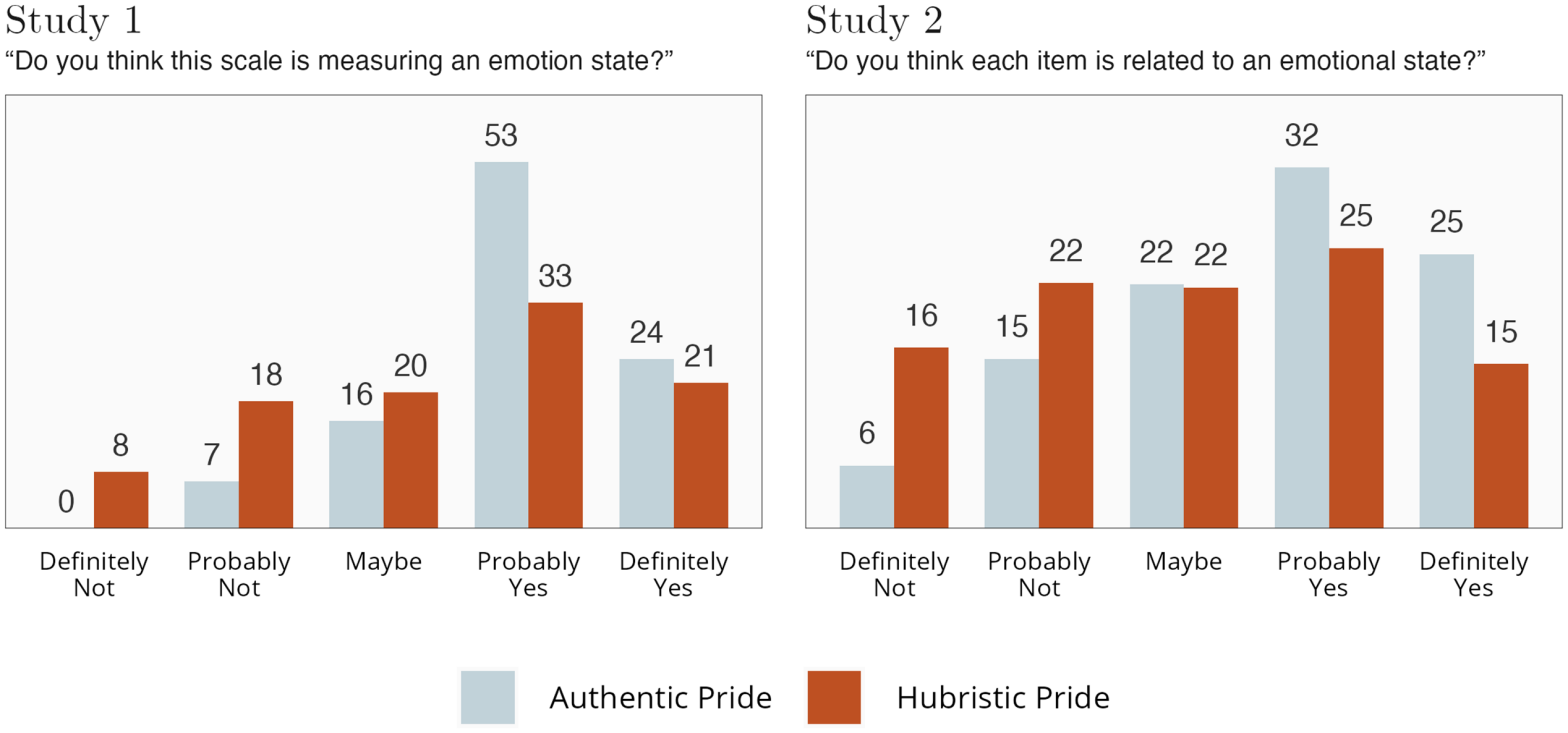
Ratings of measurement of an “emotional state”. Numbers indicate percentages.

In Studies 1 and 2, we asked participants whether they thought each scale (Study 1) or each item (Study 2) was measuring an emotional state. For Study 2, we calculated the average score for all AP items and for all HP items, and then rounded to the nearest response option. Results looked similar across studies, as can be seen in Figure 5.

#### Conclusion

Most layperson participants perceive the AP scale as probably or definitely measuring an emotional state, with few indicating it definitely or probably does not. About half of the participants seemed to perceive the HP scale as probably or definitely capturing an emotional state, but a substantial minority thought it did not.

### Positively valenced?

Pride, at least as a scientific construct, is universally discussed as a “positive emotion” (e.g., Weidman and Tracy, 2020). Dickens and Murphy (2023) pointed out that the HP scale’s scores are meta-analytically associated with negative emotion, not positive emotion, at the trait level (see Dickens and Robins, 2020). Murphy and Dickens (2023) further noted that, though the current evidence is limited, HP items are also mostly associated with negative emotion at the within-person level in experience sampling studies (Chung et al., 2022). Here we investigate whether laypersons understand these scales and items as emotionally positively valenced.

The free response data, discussed earlier above in reference to our primary question, indicated that participants overwhelmingly perceive the AP scale as related to positive emotion and not to negative emotion, but they widely differ in whether they perceive the HP scale as related to positive emotion rather than negative emotion. Here we describe additional ratings data that can supplement that information.

#### Studies 1 and 2 rating data

In Studies 1 and 2, we asked participants to consider whether someone endorsing the items “would feel good or bad about feeling that way?,” or to imagine that they endorsed the item and whether they would feel good or bad about feeling that way. This is not the same thing as asking whether someone endorsing the items “would feel good or bad;” instead, this question tries to get at what a person who is feeling AP or HP might feel *about the fact that they are feeling that way*. For example, a person who is feeling arrogant might feel sad or feel ashamed of that feeling. As a result, this question may better get at what participants are feeling when they are asked to respond to these items in a self-report questionnaire, such as when they have just had an experience and are then presented with the AP/HP scales as part of an experience sampling study.

**Figure 6:**
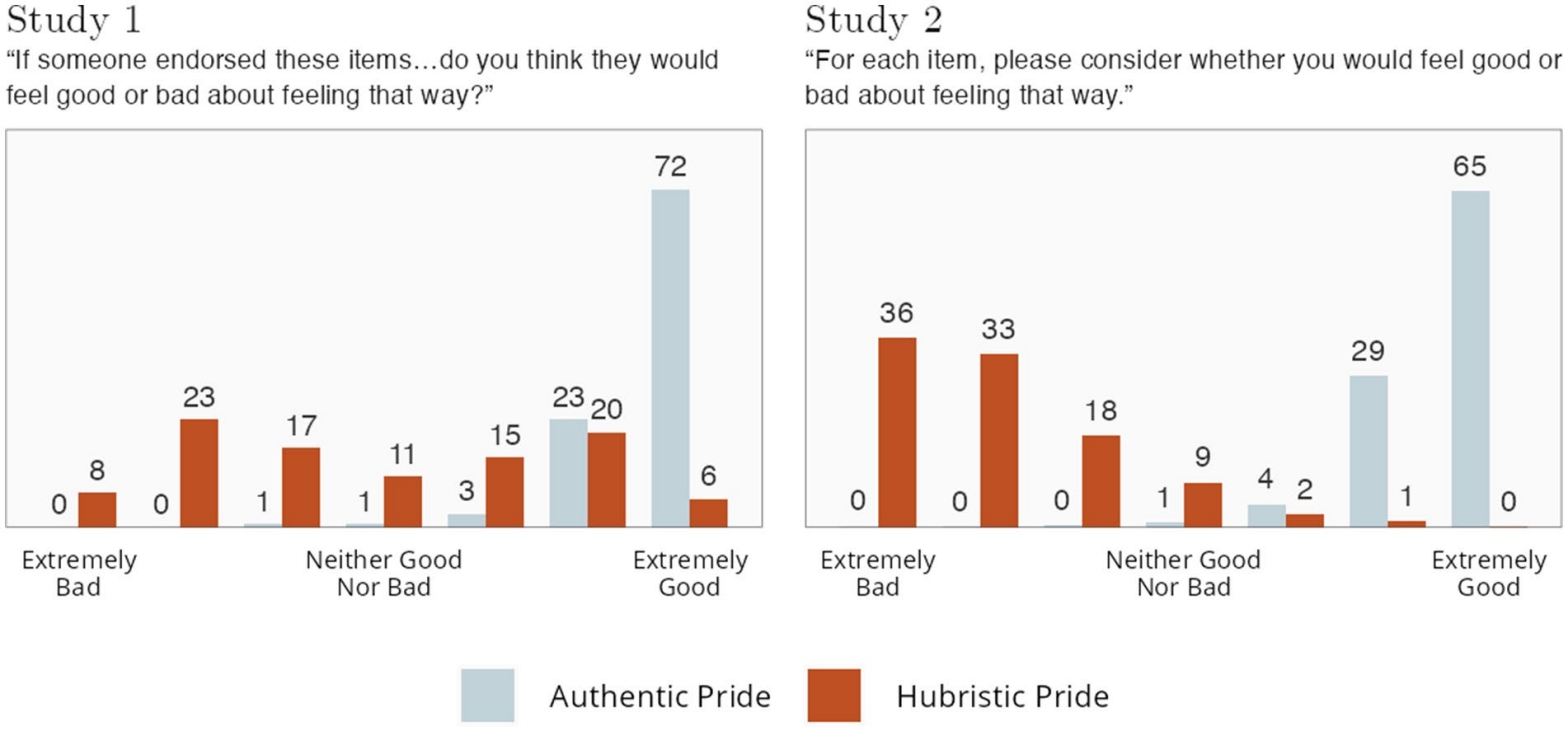
Ratings of feeling good or feeling bad about agreeing to item(s). Numbers indicate percentages.

Results are shown in Figure 6. Participants thought feeling AP would entail feeling extremely good (for both others and themselves). Participants widely differed as to whether they thought others feeling HP would feel good or bad, with roughly equal numbers indicating that others would feel bad about it as those who indicated others would feel good about it. When imagining themselves endorsing the HP items, rather than someone else, they overwhelmingly indicated they would feel bad about it. The disparity between these results appears to confirm the claim in Dickens and Murphy (2023) that the HP items are especially problematic when applying them to oneself, in comparison with applying them to others.

#### Study 3 rating data

Included in the list of other emotions they might possibly have been feeling (see Figure 2) during their AP/HP experiences were the items *good, happy,* and *content*. For AP, 98.3% of participants indicated feeling at least one of these (with 83.0% reporting all three); for HP, although 40.0% reported all three, another 37.9% reported *none* of these positive feelings. We also analyzed more general negativity items (*bad, sad, upset*). For AP, only 2.3% reported one or more of these items. For HP, 40.4% of participants reported at least one.

**Figure 7:**
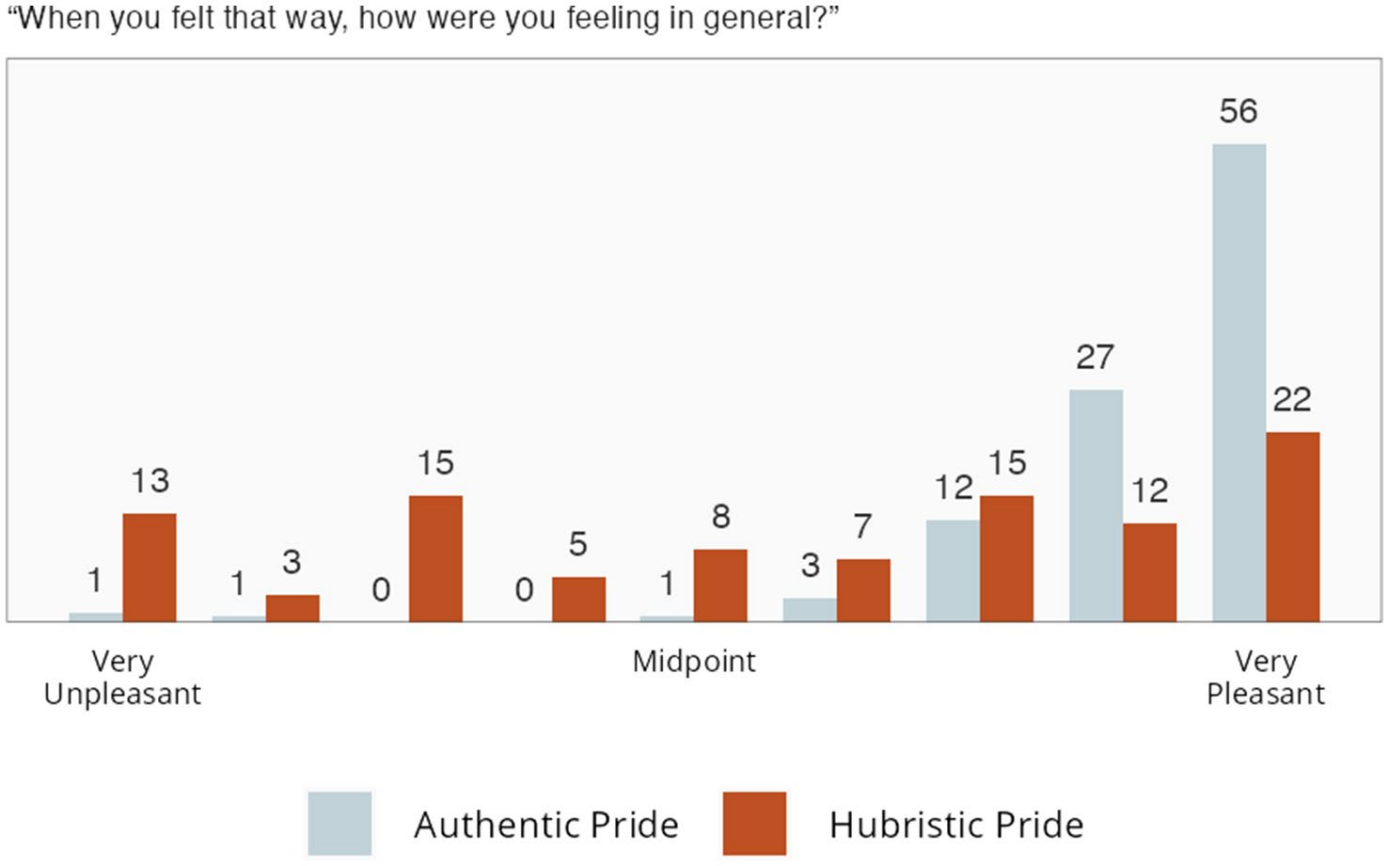
Study 3 self-assessment manikin (SAM) ratings. Numbers indicate percentages.

In addition, we had participants answer, “When you felt this way, how were you feeling in general?” using a Self-Assessment Manikin (SAM) scale of affective valence (Bradley and Lang, 1994). Participants almost universally reported AP experiences to be mostly positive. For HP experiences, though, emotional valence ranged widely across the full valence spectrum (see Figure 7).

#### Conclusion

Across all the data, whether asking people to describe and rate the scales and items, or asking them to recall their own AP or HP experiences, AP was consistently, and very strongly, related to positive emotional valence. In contrast, across the different analyses, there was no consensus across laypersons regarding HP’s emotional valence. Some participants indicated that it was emotionally negative, whereas others indicated it was emotionally positive.

### Success-related?

Of the positive emotions, pride is distinguished in that it is felt specifically in response to success in a valued domain. Dickens and Murphy (2023) argued that the AP scale items facially reference success (e.g., “like I am achieving”) but none of the HP items do so. Here we investigate whether laypersons perceive these scales/items as related to success.

#### Studies 1 and 2 rating data

In Study 1, we asked participants to consider whether they thought the scale measured something related to success. For Study 2, we asked participants to report the degree to which they thought each item sounded related to success.

**Figure 8:**
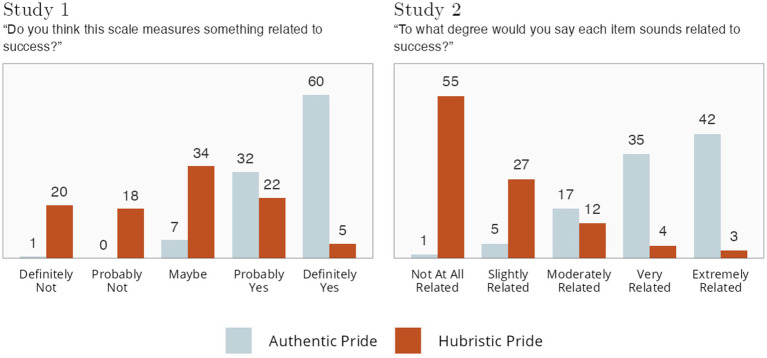
Ratings of relatedness to success. Numbers indicate percentages.

Results are shown in Figure 8. Participants generally thought the AP scale and items were strongly related to success. In contrast, participants typically thought the HP items, individually, were not much related to success; when considering the HP scale as a whole, participants widely disagreed as to whether it was measuring something related to success.

#### Study 3 free response data

For *Experience* and *Cause* free responses combined, research assistants coded whether any of a list of success-related words appeared in the response (see Supplementary materials). When describing their AP experiences, 97.8% of respondents referenced a success, versus only 52.2% of HP respondents. When discussing the cause of their AP experience, 94.5% of participants mentioned a success; only 42.4% of HP respondents mentioned a success.

#### Conclusion

Laypersons generally perceive the AP scale content as related to success. They widely disagree as to whether the HP scale is related to success.

## General discussion

Essentially all the scholars who have debated the AP and HP scales have agreed that the AP scale measures a first-person emotional experience of pride. Yet, despite a decade of debate, scholars have remained strongly divided as to whether the HP scale measures a first-person emotional experience of pride. Ample scholarly discussion and extensive empirical data have failed to produce a convergence of scholarly opinions. Recently, both those critiquing the HP scale and those defending it have pointed to an under-explored issue which could potentially bring about such convergence: how are laypersons interpreting and using the HP scale? Above all, does the HP scale align with “pride” as laypersons understand that vernacular concept?

In this article, we provide an initial, but extensive, examination of how laypersons perceive the AP and HP scales. In our first two studies, we had laypersons serve in roughly the same kind of evaluation role that panels of scholarly experts are often recruited to serve in content validity investigations: to examine scales/items and give their thoughts as to what they do and do not appear to measure. In our third study, we asked laypersons to recall a time when they felt “*successful, accomplished*, and *confident*” or a time when they felt “*pompous, conceited,* and *arrogant*,” and examined their free responses and ratings to illuminate how they are using such items when applying them to their own experiences.

As observed in our three studies, much as scholars have disagreed about the HP scale, so too do laypersons demonstrate widely differing perceptions in how they interpret the HP scale content. Although many seem to perceive it as related to “pride,” many others do not, or even associate it with theoretically opposing concepts such as shame. Although many perceive the HP scale as measuring a positive emotional state related to success, many do not, or even perceive it as relating to sadness or other negative emotions. Rather than lining up coherently with the lay concept of “pride” and/or the consensus scientific definition of pride, layperson perceptions of the HP scale appear to span a wide range of differing interpretations.

The extent of the layperson disagreement about the HP scale is brought into sharp relief by looking at laypeople’s strong agreement in their perceptions of the AP scale. Our data indicates that layperson perceptions of the AP scale largely line up with both their own understandings of the term “pride” and the consensus scientific definition of pride. Laypersons unambiguously interpret the AP content as reflecting a positive emotional state related to success. The only potential limitation of the AP scale is that it may capture a broader domain of positive self-regard and positive affect, rather than tightly capturing only pride. Indeed, we were admittedly surprised by some of the low percentages of participants spontaneously supplying the word “pride” in association with the AP items and full AP scale. However, as one reviewer pointed out, it is possible that in our attempt to reduce any bias in participant responding by referring in Study 1 to the scales simply as “personality measures” (rather than mentioning the word “emotion”), we may have inadvertently biased participants *against* supplying the emotion term “pride,” at least in response to the open question, “What do you think this scale is measuring?”.

Our results do not necessarily provide full support for all the strongest claims presented by prior critiques of the AP and HP scales (e.g., Dickens and Murphy, 2023; Holbrook et al., 2014a, 2014b; Kusano and Kemmelmeier, 2022)^18^. They do, however, clearly challenge Tracy et al.’s (2023) main remaining defense of the HP scale, which is that laypersons presumably perceive it to be measuring “pride.” Our findings show that laypersons *do not* robustly perceive it as capturing “pride.” Moreover, much as Dickens and Murphy (2023) speculated, the widely differing layperson interpretations of the HP scale demonstrate that “it is difficult to see how HP scale scores could be confidently used as an indicator of any particular construct” (p. 892).

Our practical conclusions are much the same as in prior critiques of these scales: the AP scale’s scores probably do validly assess pride, but the HP scale’s scores probably do not. While we believe the theoretical construct of hubristic pride proposed by Tracy and Robins (2004, 2007) is worthy of empirical investigation, we also think that existing research on it using the HP scale needs to be rolled back and this area of research needs to be relaunched using valid measures.

### Constraints on generality

Because “pride” is an English word, we limited participation to native English speakers, and this led to an overwhelmingly WEIRD sample. The participants were not only all English-speaking but also overwhelmingly white/Caucasian. Although the AP and HP scales were created using undergraduate samples that were also lacking in demographic diversity (Tracy and Robins, 2007), Tracy and colleagues subsequently took impressive efforts to validate their approach in other languages and with non-white populations (e.g., Shi et al., 2015). It is a very live possibility that the studies we conducted might have produced meaningfully different results and conclusions if conducted using samples focused on other demographic and cultural groups. We encourage future research to investigate the translations of the AP and HP scales and how they are being interpreted by their own native speakers (e.g., Polish adaptation, Ślaski et al., 2021; German translation, Körner and Schütz, 2023).

### Other potential limitations and future directions

There are other potential limitations of the work we have presented, but the most pressing potential limitation is that our methods and conclusions could be distorted by our own biases (we entered these studies with a negative view of the HP scale’s validity as a measure of pride). Although we tried to be conscious of this potential bias and to counter it where possible, it nonetheless posed a risk throughout our work, such as in how we constructed the surveys and in the subjective conclusions that we drew from the resulting data. We present the descriptive data here for readers to interpret for themselves.

Additionally, some researchers may prefer a very different approach to investigating the response process validity issues in this article. For example, to determine whether laypersons perceived the scales/items as reflecting “pride” as they understand that term, we focused on their free responses and found that very few participants spontaneously used that term to describe the scales/items. We chose this free response method because this is most conceptually similar to the situation faced by layperson research participants (i.e., they are not given various options as to what the scales/items might mean, but, instead, must interpret the scales/items based on themselves alone). Yet, an alternative approach could have been to, instead, present participants with a list of different concept terms (e.g., contempt, narcissism, pride, joy, anger, self-esteem, etc.) and then ask them to rate how well each scale captured each of those terms. Then, we could have observed whether “pride” was the highest rated term from among the options (e.g., is the HP scale more highly rated for “pride” than for “narcissism”?). It is a valuable open question as to whether the results and conclusions might be different when taking such an approach.

More broadly, given that this kind of lay-focused response process investigation is rare in psychology (especially beyond interview methods with a small number of people), best practices have yet to be formulated and methodological exemplars are scarce (but see very recent path-clearing work by Wolf et al., 2025). We encourage readers to critically interrogate the methods we used. We second the calls made by others (e.g., Mason et al., 2023) for expanded focus on these kinds of lay-focused investigations, and we hope that our attempts here provide valuable “grist for the mill” in developing and refining optimal methods for conducting such investigations.

We also strongly encourage researchers to study the two-facet model of pride with alternative measures, as we think it is a theory worthy of investigation. Do guilt and shame have positive self-conscious emotion parallels, as Tracy and Robins (2007) originally proposed, but the value of that original theory has been undermined by invalid measurement practices? We continue to feel that the original theoretical model should be evaluated anew with better measurements tools.

Other work could also be done regarding the hubristic pride items. Past critiques of the scales, as well as a current reviewer of the paper, have mentioned that words like arrogant and pompous may be more indicative of negative social evaluation than emotional states, and may be more easily applied to others rather than ourselves. Indeed, it appears that people are more willing to use these terms when attributing them to other people. This might suggest that these items are not necessarily capturing any internal emotional state, but are instead primarily capturing judgments we typically make regarding other people.

## Conclusion

The current work adds more evidence that the Authentic and Hubristic Pride Scales do not seem to be reflective of the theorized two-facet model of pride and arguably should not be used to measure AP and HP. While the AP scale appears to assess pride-related positive emotion in response to success, the HP scale demonstrated murkily inconsistent results for most of our analyses, both qualitative and quantitative. From our own admittedly fallible interpretation of the data, not only is the HP scale incapable of confidently assessing a layperson conception of the emotion of pride, it probably should not be used to confidently assess *any* particular psychological construct. Again, though, we strongly encourage readers to examine the distributional data from our studies and come to their own conclusions.

## Data Availability

The datasets presented in this study can be found in online repositories. The names of the repository/repositories and accession number(s) can be found below: https://osf.io/7sf6x/?view_only=fa645c24b28441c6bc10bff5d92f247c, Open Science Framework.

## Footnotes

1 "Nonetheless, critiques of the theoretical model have been raised previously, and we agree with several of these. Most notably, Holbrook et al. (2014a, 2014b) noted that extant studies do not consistently support the suggestion that AP is elicited by effort attributions and that HP is elicited by ability attributions (see Tracy and Robins, 2014). Dickens and Murphy use this point to argue that the AP/HP scales are invalid, but an equally viable interpretation is that our original model was incorrect on this particular point. In this view, AP and HP are not as analogous to guilt and shame as we initially theorized (Tracy and Robins, 2004). Although our initial hypotheses about the two facets’ distinct attributional antecedents stemmed from the speculation (first suggested by Tangney et al., 1989; see also Lewis, 2000) that a pair of positive self-conscious emotions might parallel guilt and shame, it has become clear that mapping of the parallel pairs is far from perfect” (Tracy et al., 2023, ps. 894–895).

2 Dickens and Murphy (2023) and Tracy et al. (2023) disagree as to how to frame this distinction. Dickens and Murphy (and Murphy and Dickens, 2023) argue that this means the HP scale implicitly aims to capture a “folk construct” or “folk concept,” whereas Tracy et al. object to that framing.

3 For analogy, consider that aggregating across family recipes for burritos might result in beef, beans, peppers, cheese, and wheat being listed as main ingredients. Yet, such ingredients could also be used to create quite different dishes, such as casseroles, stews, chili, etc., which the people who provided the burrito recipes would not recognize as “burrito.”

4 “Put simply, if non-experts do not understand, relate to, or accurately interpret the contents of psychological measures in a similar manner as they are believed to by experts (i.e., researchers) then psychology risks any theories and models derived from these measurements that may not accurately reflect the human mind” (Mason et al., 2023, p. 4).

5 That said, we have supplied inferential statistics in the supplementary materials for interested readers, which show empirical support that AP and HP – as measured by the AP/HP scales – are understood very differently by laypeople. We believe the more important story is about the entirety of the distributions, though, not about comparing mean differences between AP and HP.

6 For example, we also investigated whether layperson perceptions of these scales align with the two attributional pathways (e.g., effort vs. one’s own stable characteristics) in the original Tracy and Robins (2004, 2007) model. We found that they did not. Given that Tracy et al. no longer consider these attributional pathways as determinative of the scales’ validities, though, we only include this information in the supplementary materials.

7 Given that the word “prideful” has been defined as meaning “having an excessively high opinion of oneself” (Oxford languages), we considered it separately. One participant described an AP item using the word “prideful;” five participants used the word “prideful” in their descriptions of the HP items.

8 Because original independent codes were not retained, we cannot compute inter-rater reliability statistics. The present results reflect merely consensus convergence, not reliability estimation.

9 As a supplemental and somewhat exploratory analysis [see Rathje et al. (2024), preprint, for precedent], we used ChatGPT 3.5 (accessed on 8/16/2023) to extract the “three themes that were referenced by the greatest number of participants” in these free responses (see supplementary materials for the transcript of our prompts and ChatGPT output). For the AP scale, ChatGPT concluded that the three most frequently referenced themes were: self-esteem/self-worth, happiness/positive emotions, and success/achievements—relatively in line with our human coders. For the HP scale, ChatGPT concluded that the three most frequently referenced themes were: self-perception and personality traits; negative emotions and attitudes; and honesty and self-awareness. As described in the supplementary materials, when multiple iterations (five additional trials) of the ChatGPT prompts were attempted, each in a new chat, we received very consistent themes for the AP scale, with only minor changes in wording. However, we saw much more varied (and vague) themes for the HP scale over five trials. Given that human coders and ChatGPT showed similar levels of variability in themes for HP, this might indicate that HP does not reflect any specific singular construct.

10 This does not include one instance of “prideful.”

11 This includes one instance of “overjoyed.”

12 Two commonly occurring words for AP terms, not included on Weidman et al.’s list, were satisfaction (46 occurrences) and fulfillment (25).

13 This does not include eight instances of “prideful.”

14 Other commonly occurring words for HP terms were: bad (34), annoyance (28), superiority (22), guilt (22), and unhappiness (20).

15 Given the effectiveness of such relived emotion tasks in psychological research over the past few decades for numerous positive and negative emotions, it might be concerning that the majority of people struggled to think of a relevant memory for HP and might suggest that it may not be capturing a common emotional experience. It is also highly possible that the low recall for HP moments might be a result of a strong desirability bias created by the highly pejorative nature of the HP items. This is an open question that merits future investigation.

16 The contrast between low spontaneous free response use of the word pride and much higher rating of pride when presented with phrases using the word is interesting. It is possible that pride is not a high frequency word in people’s everyday life, at least in comparison with other emotion-relevant words (e.g., sad, happy), so it is less likely to be one of the first words that comes to mind for laypersons, yet, when presented with it in a sentence, laypersons understand well what it means.

17 As specified by Mercadante et al. (2021) and Tracy and Robins (2007), and most other pride theorists, “pride also requires that one attribute the cause of the success to oneself, not to an external cause” (Dickens and Murphy, 2023, p. 889). We collected data regarding that question as well, but did not include it in the definition of pride we presented to participants. As shown in the supplementary materials, though, laypersons seem to see AP items are more often internally attributed than HP items, but there was not a stark contrast between AP/HP.

18 For example, the arguments in Dickens and Murphy (2023) would predict much more agreement that a person who reported feeling “conceited” would be feeling negatively at the same time. However, across at least Studies 1 and 3, the emotion valence rating questions showed wide variability in responses for HP.
