# Supplementary material for: How do laypersons interpret the Authentic and Hubristic Pride Scales? A three-study content validity investigation
Source: Front Psychol. 2026 Feb 25;17:1699326. doi: 10.3389/fpsyg.2026.1699326 (PMC12975937; doi:10.3389/fpsyg.2026.1699326)
Supplement: Supplementary file 1 [file Supplementary_file_1.docx]

1. **Inferential Statistics, Studies 1-3**

**Table S1**

*Study 1 data (full scales)*

|  | AP | HP |  |  |  |
| --- | --- | --- | --- | --- | --- |
| Variable | *M (SD)* | *M (SD)* | *r* | *t* | *d* |
| Pride | 4.07 (.89) | 2.52 (1.20) | -.07 | 12.25*** | 1.01 |
| Emotion | 3.95 (.82) | 3.40 (1.24) | .44*** | 5.82*** | 0.48 |
| Positive Construct | 4.46 (.73) | 1.51 (.76) | .04 | 34.38*** | 2.84 |
| Negative Construct | 1.76 (1.00) | 4.54 (.68) | -.06 | 27.03*** | 2.23 |
| Success | 4.52 (.69) | 2.76 (1.17) | -.09 | 15.19*** | 1.25 |
| Effort | 4.03 (.86) | 2.29 (.95) | .10 | 17.35*** | 1.43 |
| Ability | 3.75 (.94) | 2.51 (1.02) | .16 | 11.84*** | 0.98 |
| Social Judgment | 3.52 (.97) | 4.14 (.76) | .12 | 6.50*** | 0.54 |
| *N* = 147. All variables on 5-point scales, with higher values indicating more relatedness. | | | | | |
| *** *p* < .001 |  |  |  |  |  |

**Table S2**

*Study 2 data (single-items, averaged by scale)*

|  | AP | HP |  |  | |  |  |
| --- | --- | --- | --- | --- | --- | --- | --- |
| Variable | *M (SD)* | *M (SD)* | *r* | *t* | | *d* |  |
| Pride | 4.03 (.75) | 2.67 (1.16) | -.10 | 11.58*** | | 0.94 |  |
| Emotion | 3.57 (.88) | 2.99 (1.11) | .13 | 5.31*** | | 0.44 |  |
| Positive | 4.05 (.79) | 1.30 (.45) | -.02 | 37.04*** | | 3.00 |  |
| Success | 4.11 (.64) | 1.73 (.84) | .17* | 30.38*** | | 2.47 |  |
| Effort | 3.68 (.83) | 1.49 (.62) | .12 | 27.44*** | | 2.26 |  |
| Ability | 3.66 (.89) | 1.60 (.76) | .18* | 24.00*** | | 1.94 |  |
| *N* = 153. All variables on 5-point scales, with higher values indicating more relatedness. | | | | | | | |
| * *p* < .05; *** *p* < .001 | |  |  | |  |  | |

**Table S3**

*Study 3 data (writing prompt)*

|  | AP | HP |  |  |  |
| --- | --- | --- | --- | --- | --- |
| Variable | *M (SD)* | *M (SD)* | *r* | *t* | *d* |
| Pleasant | 8.17 (1.72) | 5.61 (2.91) | .22 | 6.75*** | 0.84 |
| Social Judgment | 4.27 (.74) | 2.08 (1.15) | .09 | 13.38*** | 1.67 |
| *Note*. *N* = 64. "Pleasant" regards responses to "When you felt this way, how were you feeling in general?" from *very unpleasant* (1) to *very pleasant* (9). "Social Judgment" regards responses to "How did you think other people were feeling about you in that moment?" from *very bad about me* (1) to *very good about me* (5). | | | | | |
| *** *p* < .001 |  |  |  |  |  |

1. **Effort and Ability Attributions, Study 1**

**Effort Attributions:**

1. Question: Do you think this scale measures something **related to effort**?

**Table S4**

*Effort scores for AP and HP*

| AP POMP SCORES: | HP POMP SCORES: |
| --- | --- |
| Mode = 75 | Mode = 25 |
| SD = 21.39 | SD = 23.81 |

**Figure S1**

*Effort responses for AP and HP.*

1. Question: Do you think someone would endorse these items if they had just done well on a project they had worked really hard on?

**Table S5**

*Effort endorsements for AP and HP*

| AP POMP SCORES: | HP POMP SCORES: |
| --- | --- |
| Mode = 100 | Mode = 50 |
| SD = 13.78 | SD = 27.25 |

**Figure S2**

*Effort endorsement responses for AP and HP.*

**Ability Attributions:**

1. Question: Do you think this scale measures something **related to ability**?

**Table S6**

*Ability scores for AP and HP*

| **AP POMP SCORES:** | **HP POMP SCORES:** |
| --- | --- |
| Mode = 75 | Mode = 25 |
| SD = 23.38 | SD = 25.40 |

**Figure S3**

*Ability responses for AP and HP.*

1. Question: Do you think someone would endorse these items if they had just done well on an exam they hadn't bothered to study for? [Tracy wording]

**Table S7**

*Ability endorsements for AP and HP*

| AP POMP SCORES: | HP POMP SCORES: |
| --- | --- |
| Mode = 50 | Mode = 75 |
| SD = 26.16 | SD = 28.51 |

**Figure S4**

*Ability endorsement responses for AP and HP.*

1. **ChatGPT Prompts, Transcripts, & Interpretations, Study 1**

For Study 1 Scale Description (Free Responses):

Qualtrics Question: *From just this information alone,* ***what do you think this scale is measuring****? You are welcome to use one word or many to explain your thoughts.*

**Prompt for ChatGPT:**

I would like your help with some qualitative data analysis. I asked people to examine a short psychology questionnaire scale. Then I asked them to tell me that they think the questionnaire is measuring.

I collected their written responses to this question.

Each participant response is shown as a new paragraph (separated by a blank line). Can you tell me which three themes were referenced by the greatest number of participants?

*[copy paste AP data]*

*[repeat prompt with HP data, in new chat session]*

------------

**AP Response from ChatGPT (verbatim):**

**See transcript here:** <https://chat.openai.com/share/ed9a78fb-f3d0-40ec-a755-7c56326087f7>

Based on the participants' responses, I have identified the top three themes referenced by the greatest number of participants:

1. **Self-Esteem / Self-Worth:** Many participants mentioned that they believe the questionnaire is measuring aspects related to self-esteem, self-worth, confidence, and positive self-perception. These responses emphasize how individuals view themselves, their accomplishments, and their self-belief.

2. **Happiness / Positive Emotions:** Another prevalent theme is the measurement of positive emotions, happiness, and positive mindset. Participants noted that the questionnaire seems to assess how positively individuals feel about themselves and their achievements.

3. **Success / Achievements:** The concept of success, achievements, and self-perceived accomplishments is also a recurring theme. Participants suggested that the questionnaire is evaluating how successful individuals perceive themselves to be and how they view their achievements.

It's important to note that these themes are based on the provided responses and may involve some interpretation. If you need further analysis or assistance, please feel free to ask.

------------

**HP Response from ChatGPT (verbatim):**

**See transcript here:** <https://chat.openai.com/share/c0a7d1ff-ad39-4ef5-b6da-0cbe06d78e4a>

Based on the participants' responses, I've identified the following three themes that were referenced by the greatest number of participants:

1. **Self-Perception and Personality Traits:** This theme encompasses responses related to how individuals perceive themselves, their personality traits, and their self-worth. Many participants mentioned terms like "personality," "self-esteem," "self-worth," "self-centeredness," "narcissism," and similar concepts.

2. **Negative Emotions and Attitudes:** This theme includes responses that revolve around negative emotions, attitudes, and traits. Terms like "negativity," "negative feelings," "bad attitude," "selfishness," "arrogance," "egotism," and others fall under this theme.

3. **Honesty and Self-Awareness:** This theme refers to responses that touch upon the concepts of honesty, self-awareness, and introspection. Participants mentioned aspects like "honesty," "self-awareness," "self-perception," "negative self-reflection," and similar notions.

These three themes were referenced by the greatest number of participants in their responses about what they think the questionnaire is measuring. Keep in mind that some responses could be interpreted differently depending on context, and there might be a certain level of subjectivity in categorizing themes.

**Possible interpretations of these results:**

One thing we noted was the difference in specificity between ChatGPT’s themes for AP versus HP. Whereas the AP scale was specifically tied to self-esteem, happiness, and success, the HP scale was defined with more vague concepts like personality traits and and negative emotions and attitudes. We interpret this to possibly provide further evidence that AP is a construct more strongly tied to pride, and HP is a vague construct that could mean a wider range of things.

We also ran these same prompts through new chats over multiple iterations, to see if the themes that ChatGPT highlighted were consistent. Indeed, we found that AP was tied to the same three themes – self-esteem, happiness, and success – with slight changes in wording (e.g., achievements versus accomplishments). In contrast, the themes for HP kept changing over each iteration, ranging from “emotions and feelings,” to “comparisons and social perception,” to “narcissism/narcissistic traits.” Again, the themes often seemed vague, and often seemed negative. The inconsistency, combined with the vagueness, suggest to us further proof that the HP scale is not successfully measuring a singular construct.

This ChatGPT data could provide a new type of evidence – not biased by the human mind – suggesting that the AP scale measures a singular construct that sounds similar to pride (positive emotion relating to success and self-esteem), and that the HP scale does *not* measure an obvious singular construct. We look forward to other researchers testing this method for themselves.

1. **Internal and External Attributions, Study 2**

**Participant Instructions:** Sometimes we feel certain ways because of things we ourselves have done, and sometimes we feel certain ways because of what other people have done, or because of the situation itself. When we ourselves are the cause of an event, we say this event is "**internally attributed**." When someone or something else is the cause of an event, we say this is "**externally attributed**."

So, I might feel tired because I ran 3 miles, and this fatigue would be internally attributed; that is, I *myself* am responsible for feeling this way.

Alternatively, I might feel embarrassed because people are laughing at my mistakes, and this embarrassment would be externally attributed; that is, the *other people* laughing are the reason why I am embarrassed. (Even though I am responsible for the mistakes, it's not the mistakes themselves that cause the embarrassment, but rather the unfortunate social context of people laughing at me.)

For each item, please consider whether you think that this item might **best be INTERNALLY attributed or EXTERNALLY attributed.** If you aren't sure, you may select "Unsure."

*Response options: INTERNALLY attributed / EXTERNALLY attributed / UNSURE*

Each scale has seven items; scores therefore range from 0 to 7.

**Figure S5**

*Number of items labeled as internal attributions (Study 2).*

**Figure S6**

*Number of items labeled as external attributions (Study 2).*

1. **Weidman, Steckler, & Tracy (2017) Words Included in Study 2 Analysis**

**(Includes nouns and relevant variations)**

Anxiety

Sadness

Anger

Happiness

Fear

Disgust

Guilt

Joy
Pride

Elation

Shame
Surprise
Awe
Calmness
Gratitude
Hope
Anticipatory enthusiasm

Contentment

Interest
Jealousy

Regret

Contempt

Depression

Embarrassment

Excitement

Frustration

Irritation

Tension

Boredom

Concern

Confusion

Disappointment

Distress

Enjoyment

Envy
Inspiration

Melancholy

Nervousness
Pleasant relaxation

Relief

Shyness

Vigor

1. **Study 2 Free Response Emotion Data for AP and HP Items**

**Table S8**

*Top 20 frequently occurring terms in free responses to “What emotion would you likely be feeling for each item?” (Study 2)*

| **Authentic Pride** | |  | **Hubristic Pride** | |
| --- | --- | --- | --- | --- |
| **Term** | ***n* (%)** |  | **Term** | ***n* (%)** |
| Happiness | 450 (42.1%) |  | Sadness | 90 (8.4%) |
| Pride | 130 (12.1%) |  | Pride | 87 (8.2%) |
| Contentment | 74 (6.9%) |  | Anger | 76 (7.1%) |
| Satisfaction | 46 (4.3%) |  | Happiness | 60 (5.6%) |
| Joy | 32 (3.0%) |  | Shame | 37 (3.5%) |
| Excitement | 26 (2.4%) |  | Bad | 34 (3.2%) |
| Fulfillment | 23 (2.1%) |  | Annoyance | 28 (2.6%) |
| Confident | 21 (2.0%) |  | Confident | 27 (2.5%) |
| Successful | 16 (1.5%) |  | Arrogant | 25 (2.3%) |
| Accomplished | 15 (1.4%) |  | Guilt | 22 (2.1%) |
| Pleased | 15 (1.4%) |  | Superiority | 22 (2.1%) |
| Good | 9 (.8%) |  | Satisfaction | 18 (1.7%) |
| Sadness | 9 (.8%) |  | Embarrassment | 16 (1.5%) |
| Energetic | 7 (.7%) |  | Conceited | 15 (1.4%) |
| Motivated | 7 (.7%) |  | Insecure | 14 (1.3%) |
| Relief | 7 (.7%) |  | Cocky | 13 (1.2%) |
| Complete | 6 (.6%) |  | Rude | 12 (1.1%) |
| Anticipatory enthusiasm | 5 (.5%) |  | Disgust | 11 (1.0%) |
| Hope | 5 (.5%) |  | Fear | 11 (1.0%) |
| Secure | 5 (.5%) |  | Selfish | 11 (1.0%) |

1. **Related Terms Included in Analyses Across Studies**

**Success-Related Terms**

Success-/Succeed-

Accomplish-

Achiev-

Winning/Winner

Promoted/Promotion

Task/job completion

Reached Goal

Champion

**not included: productive/producing, satisfied, complete, fulfilled, efficient, motivated*
